# Predictive Value of First Amniotic Sac IL-6 and Maternal Blood CRP for Emergency Cerclage Success in Twin Pregnancies

**DOI:** 10.3390/jpm15010037

**Published:** 2025-01-19

**Authors:** Diana María Diago-Muñoz, Alicia Martínez-Varea, Ricardo Alonso-Díaz, Alfredo Perales-Marín, Vicente José Diago-Almela

**Affiliations:** 1 Department of Obstetrics and Gynecology, General University Hospital, 46014 Valencia, Spain; diago_dia@gva.es; 2 Department of Obstetrics and Gynecology, La Fe University and Polytechnic Hospital, 46026 Valencia, Spain; perales_alf@gva.es (A.P.-M.); diago_vicalm@gva.es (V.J.D.-A.); 3 Department of Medicine, CEU Cardenal Herrera University, 12006 Castellón de la Plana, Spain; 4 Faculty of Health Sciences, Universidad Internacional de Valencia, 46002 Valencia, Spain; 5 Department of Clinical Laboratory, La Fe University and Polytechnic Hospital, 46026 Valencia, Spain; alonso_ricdia@gva.es; 6 Department of Pediatrics, Obstetrics and Gynecology, Faculty of Medicine, University of Valencia, 12006 Valencia, Spain

**Keywords:** cervical insufficiency, physical examination-indicated cerclage, emergency cerclage, bulging membranes, twin pregnancy, amniocentesis, IL-6, CRP

## Abstract

**Objectives:** To assess the usefulness of first amniotic sac Interleukin-6 (IL-6) to rule out intra-amniotic inflammation (IAI), as well as maternal blood c-reactive protein (CRP), to select patients with a twin pregnancy who may benefit from an emergency cerclage. **Materials and Methods**: Retrospective, descriptive study among all patients with a twin pregnancy and mid-trimester bulging membranes admitted to a tertiary Hospital from January 2012 to September 2023. According to the Hospital’s Protocol, all patients received a vaginal and abdominal ultrasound, a maternal blood test, and an amniocentesis of the first sac to rule out IAI, defined by IL-6 ≥ 2.6 ng/dL. **Results:** A total of 28 patients with a twin pregnancy and mid-trimester bulging membranes were included. Among them, 18 patients (64.28%) had IL-6 levels ≥ 2.6 ng/dL. Cerclage was placed in 10 patients with IL-6 < 2.6 ng/dL. Perinatal mortality in pregnancies with IL-6 ≥ 2.6 ng/dL was 77.22%. The gestational age at delivery of patients with IL-6 < 2.6 ng/dL was 34 ± 3 weeks, compared to 23 ± 4 weeks when IL-6 was ≥2.6 ng/dL (*p* < 0.001). The latency to delivery with IL-6 < 2.6 ng/dL was 88.1 ±31.56 days, compared to 13.11 ± 20.43 days when IL-6 was ≥2.6 ng/dL (*p* < 0.001). Significant differences were found in maternal blood CRP levels in both study groups (no IAI 4.32 ± 3.67 vs. IAI 13.32 ± 15.07, *p* < 0.05). The area under the curve with an ROC curve was 0.799 (IC 95% 0.596–0.929), with a cut-off of 3.9 mg/L (S 94.4%, % E 62.5%). The gestational age at delivery with CRP < 3.9 mg/L was 33 ± 5 weeks, while in cases with CRP ≥ 3.9 mg/L, it was 24 ± 5 weeks (*p* < 0.001). The latency days to delivery were 86.5 ± 44.88 and 21.95 ± 30.97 days (*p* < 0.01), respectively. A positive correlation between the IL-6 values of both amniotic sacs was obtained, along with the Spearman coefficient correlation rank (rho = 0.835, *p* < 0.001). **Conclusions:** Compared to those with IAI, patients with a twin pregnancy and mid-trimester bulging membranes without IAI who underwent emergency cerclage had a significantly higher interval from diagnosis to delivery, as well as a significantly lower incidence of preterm birth < 34 weeks and perinatal death. Further studies are needed to assess whether the IL-6 of the first amniotic sac and maternal blood CRP might constitute a useful parameter to select patients who may benefit from an emergency cerclage.

## 1. Introduction

Compared to singleton pregnancies, multiple gestations are associated with a higher risk of perinatal morbidity and mortality, particularly due to the elevated incidence of preterm birth (PTB) [1,2]. The incidence of PTB is 59% before 37 and 11% before 32 weeks of gestation [3]. The onset of PTB in twin pregnancies is mainly spontaneous, followed by the preterm premature rupture of membranes. However, a significant proportion of these pregnancies are delivered preterm after iatrogenic-indicated obstetrical interventions [3].

Cervical insufficiency is a well-known risk factor for preterm birth (PTB) in both singleton and twin pregnancies [4,5,6,7]. When cervical dilatation and bulging membranes in the second trimester are demonstrated, treatment options include expectant management or the placement of an emergency or a physical examination-indicated cerclage. The available literature has revealed that the emergency cervical cerclage is an effective treatment option for singleton [8,9] and twin pregnancies [10,11,12,13,14,15] with cervical insufficiency. Nonetheless, the optimal gestational age for cervical cerclage placement and the effectiveness of emergency cervical cerclage in multiple pregnancies remain controversial [16].

Intra-amniotic inflammation/infection constitutes a cause of cervical insufficiency, and its presence is a risk factor for adverse outcomes. The outcome of patients with microbiologically proven intra-amniotic infection is similar to that of patients with intra-amniotic inflammation and a negative amniotic fluid culture. Amniotic fluid interleukin-6 (IL-6) ≥ 2.6 ng/mL without positive amniotic culture indicates intra-amniotic inflammation (IAI) [17,18]. Additionally, maternal blood inflammatory markers such as c-reactive protein (CRP) are associated with PTB [19], and high levels of CRP have been found in patients with the preterm premature rupture of membranes and intra-amniotic inflammation/infection [20].

Thus, this study aimed to assess the usefulness of first amniotic sac IL-6 to rule out IAI, and then maternal blood CRP, to select patients with a twin pregnancy who may benefit from an emergency cerclage.

## 2. Materials and Methods

### 2.1. Study Design and Participants

This is a retrospective, descriptive study among all patients with a twin pregnancy and mid-trimester bulging membranes admitted to a tertiary Hospital from January 2012 to September 2023. The study was approved by the Ethics Committee of La Fe University and Polytechnic Hospital (protocol code 2020-271-1 and date of approval 29 April 2020). Informed consent was obtained from all patients. The presence of bulging membranes was defined as asymptomatic cervical dilation of the external os (≥1 cm with prolapse of membranes at or beyond the external os) by physical examination in the absence of uterine contractions, or prolapsed membranes detected by physical examination in the absence of uterine contractions [11].

All pregnant women with a twin pregnancy and bulging membranes in the second trimester of pregnancy who were admitted to La Fe University and Polytechnic Hospital, Valencia, Spain, from January 2012 to September 2023, were included. The exclusion criteria were the following: uterine contractions, clinical chorioamnionitis [21,22], premature rupture of membranes, active bleeding, cervical dilatation > 3 cm, fetal congenital anomaly, fetal death, <14^+0^ weeks gestation, >26^+6^ weeks gestation, and no signature of informed consent by the pregnant woman.

According to the hospital’s protocol [23], patients with bulging membranes received physical examination as well as vaginal and abdominal ultrasonography at the time of the hospital admission. Additionally, a blood test to analyze inflammatory markers such as leukocytes and CRP was performed on all pregnant patients. All patients underwent an amniocentesis of the first amniotic sac (only in the 12 first cases were both amniotic sacs punctured) with a 20-gauge 15 cm-long needle and a 10 cc aspiration syringe. Within the hospital’s protocol, 70 ccs of amniotic fluid were extracted, 10 ccs for microbial analysis, 10 ccs for biochemical analysis (IL-6, leukocytes, glucose, leukocyte esterase, lactate dehydrogenase (LDH), and procalcitonin), and an additional 50 cc for amnioreduction. Amnioreduction was performed according to the hospital’s protocol, despite the absence of a polyhydramnios diagnosis. It was performed given that amnioreduction could benefit those patients who subsequently may take advantage of a cervical rescue cerclage [24,25,26]. IAI was defined by amniotic IL-6 > 2.6 ng/mL [18]. Intraamniotic infection was determined by the presence of a positive bacterial culture [21].

Cerclage was placed in 10 cases after ruling out IAI. The other 18 cases were treated with bed rest, subcutaneous low-molecular-weight heparin 40 mg/24 h, and intravenous antibiotics (metronidazole 500 mg/8 h, ceftriaxone 1 g/24 h and clarithromycin 500 mg/8 h).

### 2.2. Assays

Levels of IL-6 in the amniotic fluid were measured with a commercially available lateral flow immunoassay designed for the quantitative measurement of human IL-6. Interpretation by Milenia POCScan Reader or PicoScan Systems (MileniaBiotec, Gieben, Germany). The laboratory results were shown within ninety minutes after the extraction [18]. A blood test to assess inflammatory markers, including leukocyte count and CRP, was performed for all pregnant patients upon hospital admission.

### 2.3. Cervical Cerclage Placement

The results of the amniotic fluid parameters were usually reported within 2 h, and the cerclage was performed within the first 24 h after the diagnosis. McDonald or Shirodkar cerclage techniques were used according to the surgeon’s decision at the moment of the procedure. Under spinal anesthesia, prolapsed fetal membranes were replaced with an inflated Foley catheter (14 F, 30-cc balloon; Yushin Medical Co., Seoul, Republic of Korea), and then cerclage was performed using 5 mm Mersilene tape (Ethicon Inc., Aneau, France). Ten trained practitioners performed the procedure at the tertiary La Fe University and Polytechnic Hospital. Before or immediately after surgery, 2 g intravenous augmentin and 100 mg suppository of indomethacin were administered.

### 2.4. Statistical Analysis

The comparative study used non-parametric tests, the Mann–Whitney U test for quantitative variables and the Kruskal–Willis test for categorical variables. The kappa concordance index was also used to evaluate the agreement of different parameters.

To determine the clinical utility of the CRP in the prediction of delivery, ROC analysis was used to determine the AUC of CRP that would correctly classify the maximum number of participants. After obtaining a cut-off (Youden’s index), the sensitivity, specificity, and positive and negative likelihood ratios (LR) were calculated.

The Kaplan–Meier curve was used for the survival analysis, and Log-Rank tests were applied for Kaplan–Meier curve comparisons. SPSS 25 (IBM, Chicago, IL, USA) was used for statistical analysis. A *p*-value < 0.05 was considered significant.

## 3. Results

A total of 28 twin pregnancies were included in the study. All patients showed a cervical dilatation between 1 and 3 cm. The characteristics of the 28 twin pregnancies included in the study are shown in Table 1. The average age of patients was 34 ± 5 years. A total of 26 (92.85%) women were nulliparous. The mean gestational age at diagnosis of bulging membranes was 21 (±2.5) weeks. Unfortunately, 18 (64.28%) patients had amniotic IL-6 ≥ 2.6 ng/dL. Cerclage was placed in 10 cases, all with amniotic IL-6 < 2.6 ng/dL, approximately one-third of all included patients. The McDonald cerclage technique was performed in six patients, and the Shirodkar type was placed in four patients. No complications were observed during the cerclage or in the postoperative period. There were six cases with a positive culture and IL-6 > 2.6 ng/dL, as shown in Table 2.

There were two cases with a positive culture and IL-6 < 2.6 ng/dL (bacterial colonization), as shown in Table 3. The isolated bacteria were *Klebsiella oxytoca* and *Staphylococcus* coagulase-negative, respectively. In the first case, we performed a McDonnald-type cerclage at 15 weeks and 3 days of pregnancy, and the patient delivered at 36 weeks and 6 days (150 days latency to delivery). The newborns’ weights were 2275 g and 2600 g. The other patient was diagnosed with bulging membranes at 22 weeks. She underwent a McDonnald-type cerclage and started with uterine contractions at 35 weeks and 5 days of pregnancy. An elective C-section due to the first fetus showing breech presentation was performed (96 days of latency to delivery). The newborns’ weights were 2125 g and 2700 g.

Pregnancy outcomes are shown in Table 4. The perinatal mortality in pregnancies with IL-6 ≥ 2.6 ng/dL was 77. 22%. In the group of patients with amniotic IL-6 < 2.6 ng/dL, the survival of the newborns was 100%, and the newborns’ weights ranged from 1120 g to 2760 g.

**Figure 1:**
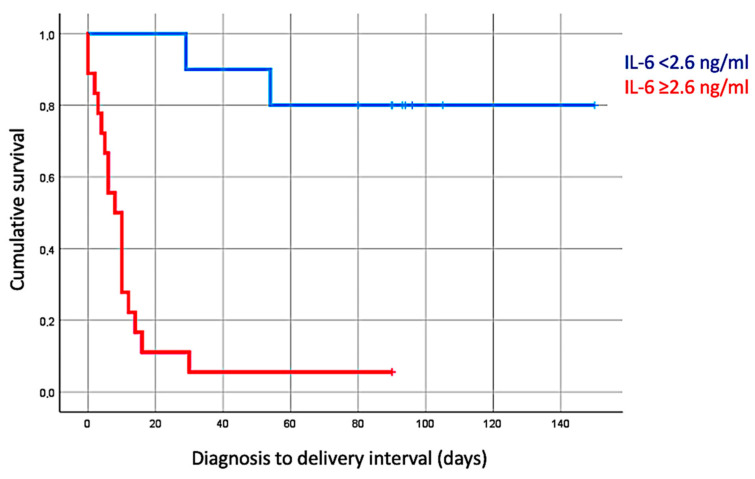
Survival of patients with and without intra-amniotic inflammation (log-rank 0.001).

The average gestational age at delivery with IL-6 < 2.6 ng/dL was 34 ± 3 weeks, compared to 23 ± 4 weeks (*p* < 0.001) in the group with amniotic IL-6 ≥ 2.6 ng/dL. The latency to delivery days with IL-6 < 2.6 ng/dL was 88.1 (±31.56) days, compared to 13.11 ± 20.43 days (*p* < 0.001) in the group with amniotic IL-6 ≥ 2.6 ng/dL. The latency to delivery estimation with IL-6 < 2.6 ng/dL was 18 weeks and 2 days (log-rank 0.001), as shown in Figure 1.

Significant differences were seen in maternal blood CRP when comparing patients with amniotic IL-6 < 2.6 ng/dL and those with IL-6 ≥ 2.6 ng/dL. The area under the curve was calculated based on the ROC curve 0.799 (IC 95% 0.596–0.929), finding a cut-off of 3.9 mg/L (Se 94.4%, Sp 62.5%), a positive LR of 2.52, and a relevant negative LR of 0.089.

The average gestational age at delivery when CRP was <3.9 mg/L was 33 ± 5 weeks, and in cases of CRP ≥ 3.9 mg/L, it was 24 ± 5 weeks (*p* < 0.001), as shown in Table 5. The latency days were 86.5 ± 44.88 days vs. 21.95 ± 30.97 days (*p* < 0.01), respectively. Only one patient had CRP < 3.9 mg/L with IL-6 ≥ 2.6/mL. She delivered at 23 weeks and 3 days, with 10 latency days from diagnosis to delivery. Three patients had CRP ≥ 3.9 mg/L with IL-6 < 2.6 ng/dL. These three patients delivered at 26 weeks, 29 weeks and 4 days, and 37 weeks, respectively, with 29, 90, and 94 latency days from diagnosis to delivery, respectively.

Maternal blood CRP was calculated in all patients except two patients due to a laboratory error. In the first case, the IL-6 value in the first amniotic sac was 0.13 ng/dL, and in the second sac it was 0.09 ng/dL; the amniotic fluid culture was negative in both sacs, and the maternal blood leukocytes were 8510/mm^3^. A McDonald-type cerclage was performed on day 153, and delivery occurred on day 258. The time from diagnosis to delivery was 105 days. The patient underwent a cesarean section, and the neonatal weights were 2760 g and 2680 g. In the second case, the IL-6 of the first amniotic sac was 2.14 ng/dL, the first amniotic sac amniotic fluid culture was negative, and the maternal blood leukocytes numbered 13,010/mm^3^. A Shirodkar-type cerclage was performed on day 174, and delivery was undertaken on day 238. The time from diagnosis to delivery was 54 days. A preterm premature rupture of membranes was diagnosed at 29 + 2 weeks. The patient underwent a cesarean section, and the neonatal weights were 1820 g and 1760 g.

A positive correlation was obtained between the IL-6 values of both the first and the second amniotic sacs, with Spearman coefficient correlation ranks (rho = 0.853, *p* < 0.001). There were two cases with IL-6 ≥ 2.6 ng/dL in the first amniotic sac and IL-6 < 2.6 ng/dL in the second. The amniotic fluid culture was, in both cases, negative. The agreement between qualitative IL-6 in both sacs was determined. The kappa index was 0.612, *p* = 0.001, which implies a substantial agreement between both parameters.

## 4. Discussion

Compared to those with IAI, patients with a twin pregnancy and mid-trimester bulging membranes without IAI who underwent emergency cerclage had significantly higher intervals from diagnosis to delivery and newborn weights, and significantly lower incidences of preterm birth < 34 weeks and perinatal death. Additionally, maternal CRP might constitute an additional parameter to help select those patients who may benefit from emergency cerclage.

It has been reported that, in pregnancies with bulging membranes, fetal outcomes significantly improve when cerclage is placed [27]. In addition, in pregnancies with bulging membranes that undergo an emergency cervical cerclage, there are no differences regarding survival between singleton and twin gestations [15]. Thus, emergency cerclage should be considered in both singleton and twin pregnancies.

Zeng et al. [28] stated that emergency cerclage in women with cervical insufficiency was associated with an overall 40% decrease in spontaneous PTB before 28 weeks, and a prolongation of latency to delivery by 5 weeks. The strongest predictor of spontaneous PTB before 28 weeks after cerclage was a positive cervical culture. The presenting sac is expected to display predominant involvement in intra-amniotic inflammation/infection because the ascension from the lower genital tract is the primary pathway for intra-amniotic infection [17]. Interestingly, Kyung Joon Oh et al. have observed that the presence of IAI in both amniotic sacs occurred in 63% (22/35) of cases. Of the remaining 13 cases, the inflammatory process was detectable in the presenting sac, except for in one case [29]. The present study has obtained a positive correlation between the IL-6 values of both amniotic sacs. All cases with IL-6 ≥ 2.6/mL in the first amniotic sac also displayed IL-6 ≥ 2.6/mL in the second. Only two cases explained in the results section displayed an IL-6 ≥ 2.6/mL in the first sac and an IL-6 < 2.6/mL in the second sac. The outcomes of both gestations were adverse, the same as when IL-6 was ≥2.6/mL in both sacs. With these findings, it can be concluded that it might be optimal to only perform amniocentesis in the first sac, given that this is the one that is more exposed to an abnormal milieu, and the one that will determine the prognosis of the gestation.

It has been well-documented that emergency cerclage is associated with poor outcomes in the presence of IAI or microbial invasion of the amniotic cavity [30]. In the present study, we aimed to identify twin pregnancies with bulging membranes that might benefit from emergency cervical cerclage by ruling out IAI. For this purpose, IL-6 levels were determined in the first amniotic sac, and patients diagnosed with IAI were managed with antibiotic therapy. Our group has previously demonstrated that IL-6 levels in amniotic fluid may serve as a valuable biomarker for guiding the management of patients with singleton pregnancies and bulging membranes, specifically for the placement of rescue cerclage [23]. Recent studies have suggested that rescue cerclage, combined with macrolide antibiotic therapy, may improve pregnancy and neonatal outcomes in cases of singleton pregnancy and cervical insufficiency complicated by IAI when compared with expectant management. These findings highlight the potential benefit of emergency cerclage placement in singleton pregnancies with bulging membranes even in the context of IAI [31]. Further research is required to evaluate whether combining emergency cervical cerclage with antibiotic treatment in patients with a singleton or a twin pregnancy with bulging membranes and IAI can effectively prolong pregnancy and improve pregnancy outcomes when compared with antibiotic treatment alone.

CRP is an acute-phase reactant protein primarily induced by the IL-6 action of the gene responsible for the transcription of CRP during the acute phase of an inflammatory/infectious process. There is a correlation between increasing levels of IL-6 during inflammations and increasing levels of CRP [32]. The determination of this correlation is used as a tool to define the presence and degree of the inflammatory–infectious process, conditioning diagnostic and therapeutic attitudes [33]. CRP has been used as a prognostic factor in acute infections. Shim SS et al. observed that a vaginal fluid CRP cut-off of 10 ng/mL had a specificity of 89% and a sensitivity of 45% in the identification of IAI [34]. Patients with vaginal fluid CRP concentrations of >10 ng/mL had higher rates of preterm delivery within five days, funisitis, and histologic chorioamnionitis than those with a vaginal fluid CRP concentration below this cut-off 30. A higher cut-off in the maternal serum of CRP (17.5 mg/L) was established to identify IAI with similar specificity (96%) and sensitivity (47%) [20].

Significant differences have been seen in maternal blood CRP in the present study. Preoperative CRP values < 3.9 mg/dL showed a major survival. The same cut-off was published by Minakami et al. They observed that a preoperative CRP value ≤ 4.0 mg/dL, a WBC count ≤ 14,000/microL, and cervical dilatation ≤ 4.0 cm were significantly associated with the prolongation of pregnancy after an emergency cervical cerclage [35]. Additionally, it was stated in 2017 that elevated peripheral C-reactive protein levels ≥ 0.4 mg/dL (aOR: 0.34; 95% CI: 0.180.65) are associated with a significantly reduced likelihood of reaching 28 weeks’ gestation before delivery in pregnant women with bulging membranes [36]. A recent study stated that the stronger predictors of spontaneous PTB before 28 weeks after an emergent cervical cerclage were pre-operation WBC > 11.55 × 10^9^/L, CRP > 10.1 mg/dL, and cervical dilation > 3.5 cm [37]. Other studies support this [38]. In the present study, a substantial agreement between CRP and IL-6 has been found. Thus, CRP could help to select those patients who may benefit the most from an emergency cerclage placement when amniocentesis cannot be performed, or the analysis of amniotic fluid parameters cannot be carried out. Nonetheless, further studies are needed to assess whether maternal blood CRP might constitute a useful parameter to select patients who may benefit from an emergency cerclage.

Another important finding of this study is the observation of amniotic colonization, defined as a positive amniotic culture with IL-6 < 2.6 ng/dL [39]. It has been described that those pregnant patients with amniotic colonization had similar outcomes to those with negative fluid, defined by IL-6 < 2.6 ng/dL [39,40]. This finding has also been observed in the present study. There were two cases with a positive culture and IL-6 < 2.6 ng/dL, indicative of bacterial colonization. Both cases underwent an emergency cervical cerclage, and delivered at 36 weeks and 6 days (with a latency of 150 days) and at 35 weeks and 5 days (96 days of latency to delivery), respectively. Thus, a positive amniotic fluid culture with IL-6 < 2.6 ng/mL, also known as early colonization [39,40], did not adversely impact the progression or prognosis of these two pregnancies. Previous reports have indicated that the isolation of microorganisms from amniotic fluid in the absence of IAI is indicative of a benign condition [39,40] most likely reflecting contamination during the specimen collection or laboratory processing, rather than early colonization or infection [40].

Regarding the type of suture used for the emergency cerclage, a study published in 2016 [41] states that a braided suture induces a persistent shift towards dysbiosis of the vaginal microbiome, characterized by a reduction in *Lactobacillus* spp. and the enrichment of pathogens causing premature cervical remodeling. We decided to continue using a Mersilene suture for both types of emergency cerclage, McDonald and Shirodkar, as a subsequently published study [42] has shown no significant differences in maternal infection rates and adverse neonatal outcomes. There is also no difference in the prolongation of pregnancy when comparing the different suture materials used for the indicated locks.

The present study was not designed to compare the outcomes of different types of emergency cerclage (McDonald vs. Shirodkar). Furthermore, the limited sample size precluded any such comparative analysis. Future research may focus on evaluating pregnancy outcomes according to the specific type of emergency cerclage performed.

Concerning previously published series, the principal strength of our work is that it improves the current knowledge on treating cervical insufficiency in twin gestations. Importantly, this study is one of the few available studies providing data supporting the assessment of intra-amniotic IL-6 exclusively from the first amniotic sac in twin pregnancies with bulging membranes. Additionally, it is one of the few studies to explore the potential usefulness of maternal blood CRP as a non-invasive alternative to amniocentesis, which is an invasive procedure. The primary limitation of this study is the limited sample size, attributable to the low incidence of the pathology. Additionally, according to the Hospitals’ Protocol, all patients without intra-amniotic inflammation or infection underwent emergency cervical cerclage. This protocol inherently precluded the inclusion of a comparison group of patients without intra-amniotic inflammation or infection who did not receive a cerclage, which could have allowed for a direct analysis of the impact of emergency cerclage in these cases. Nonetheless, comparing the outcomes of cerclage placement versus no cerclage in patients with twin pregnancies and bulging membranes without intra-amniotic inflammation or infection raises ethical challenges, given the current clinical consensus strongly favoring cerclage in such scenarios.

## 5. Conclusions

In the present study, compared to those with IAI, patients with a twin pregnancy and mid-trimester bulging membranes without IAI who underwent emergency cerclage had a significantly longer diagnosis-to-delivery interval, higher mean gestational age at delivery, higher mean birth weight, and a significantly lower incidence of preterm birth < 34 weeks and perinatal death. Further studies are needed to assess whether maternal blood CRP might constitute a useful non-invasive tool to select patients who may benefit from an emergency cerclage.

## Figures and Tables

**Table 1:** Basal characteristics, maternal blood inflammatory markers, and amniotic fluid parameters studied in patients with a twin pregnancy and bulging membranes in the second trimester, according to the presence or absence of intra-amniotic inflammation. Data are shown as mean and standard deviation or percentage.

	IL-6 < 2.6 ng/mL (n = 10)	IL-6 ≥ 2.6 ng/mL (n = 18)	*p*
Average age (years)	34.6 ± 5.7	33.94 ±8.55	NS
Nulliparous	9 (90%)	17 (94.44%)	NS
Assisted reproductive techniques	4 (40%)	10 (55.6%)	NS
Previous miscarriage	2 (20%)	4 (22.2%)	NS
Gestational age at diagnosis (weeks)	21^2/7^ ± 3	21^6/7^ ± 2	NS
Blood leukocyte (/mm^3^)	10,684 ± 2807	12,168 ± 2948	NS
Blood CRP (mg/dL)	4.32 ± 3.67	13.32 ± 15.07	<0.05
Blood neutrophils (%)	78.5 ± 11.91	77.40 ± 7.34	NS
Blood fibrinogen (mg/dL)	627.4 ± 71.17	624.29 ± 78.74	NS
Amnionic glucose (mg/dL)	30.90 ± 19.63	22.61 ± 9.71	NS
Amnionic leukocyte (/µL)	69.4 ± 95.15	246 ± 693.32	NS
Amniotic LDH (U/L)	200 ± 85.88	576.6 ± 698.39	=0.058
Amniotic procalcitonin (ng/mL)	0.058 ± 0.032	0.042 ± 0.014	NS
Amniotic positive leukocyte esterase	1/7 (14.2%)	5/14 (35.7%)	NS
Positive culture amniotic fluid	2 (20%)	6 (33.3%)	NS

NS: not significant.

**Table 2:** Patients with IL-6 > 2.6 ng/dL and positive culture. All patients with intraamniotic infection and/or inflammation were treated with intravenous metronidazole 500 mg/8 h, intravenous ceftriaxone 1 g/24 h, and oral clarithromycin 500 mg/8 h for 7 days (if delivery does not occur earlier), whether the culture was positive or not. If the antibiogram showed resistance to any antibiotic, it was changed, although in our case, this was unnecessary.

Case	Bacteria	Treatment	Time of Delivery
Case 1	*Streptococcus viridans* in both amniotic sacs	Metronidazole 500 mg/8 h, ceftriaxone 1 g/24 h and clarithromycin 500 mg/8 h 6 days.	Delivery 6 days after diagnosis.
Case 2	*Ureaplasma* spp. in both amniotic sacs	Metronidazole 500 mg, ceftriaxone 1 g and clarithromycin 500 mg (only 1 dose).	Delivery the same day of diagnosis.
Case 3	*Streptococcus sanguis* in the first amniotic sac	Metronidazole 500 mg/8 h, ceftriaxone 1 g/24 h and clarithromycin 500 mg/8 h 5 days.	Delivery 5 days after diagnosis.
Case 4	*Ureaplasma* spp. in the first amniotic sac	Metronidazole 500 mg/8 h, ceftriaxone 1 g/24 h and clarithromycin 500 mg/8 h 12 days.	Delivery 12 days after diagnosis.
Case 5	*Streptococcus agalactiae* in the first amniotic sac	Metronidazole 500 mg, ceftriaxone 1 g and clarithromycin 500 mg (only 1 dose).	Delivery the same day as diagnosis.
Case 6	*Fusobacterium nucleatum* in the first amniotic sac	Metronidazole 500 mg/8 h, ceftriaxone 1 g/24 h and clarithromycin 500 mg/8 h 3 days.	Delivery 3 days after diagnosis.

**Table 3:** Two patients underwent cervical cerclage (IL-6 < 2.6 ng/dL) and had a positive amniotic fluid culture.

Cervical Cerclage (n = 10): Two Cases with Positive Amniotic Fluid Culture	Amniotic Fluid Culture	Gestational Age Cerclage Placement	Gestational Age and Mode of Delivery	Latency to Delivery (Days)	Newborns’ Weights
Case 1	*Klebsiella oxytoca*	McDonald cerclage at 15^+3^ weeks	Vaginal delivery at 36^+6^ weeks	150	2275 g and 2600 g
Case 2	*Staphylococcus* coagulase-negative	McDonald cerclage at 22^+0^ weeks	Cesarean section at 35^+5^ weeks due to regular uterine contractions and first fetus with breech presentation	96	2125 g and 2700 g

**Table 4:** Pregnancy outcomes in patients with and without intra-amniotic inflammation.

	IL-6 < 2.6 ng/mL (n = 10)	IL-6 ≥ 2.6 ng/mL (n = 18)	*p*
Gestational age at delivery (weeks)	34^1/7^ ± 3	23^5/7^ ± 4	<0.001
Diagnosis-to-delivery interval (days)	88.10 ± 31.56	13.11 ± 20.42	<0.001
Maternal hospital admission (days)	6.60 ± 5.25	8.44 ± 5.32	NS
First newborn birth weight (g)	2008.8 ± 656.26	677.72 ± 505.02	<0.001
Second Newborn birth weight (g)	1992 ± 727.12	664.22 ± 498.92	<0.001
Preterm birth (<34 weeks)	3 (30%)	17 (94.44%)	<0.001
Perinatal death	0	13 (72.22%)	<0.001

NS: not significant.

**Table 5:** Pregnancy outcomes of patients with a twin pregnancy and bulging membranes in the second trimester, according to maternal blood CRP. Two patients lacked data regarding CRP.

	CRP < 3.9 ng/L (n = 8)	CRP ≥ 3.9 ng/L (n = 18)	*p*
Average gestational age at delivery (weeks)	33 ± 5	24 ± 5	<0.001
Diagnosis-to-delivery interval (days)	87 ± 45	22 ± 31	<0.01
Notes	One patient CRP < 3.9 mg/L and IL-6 ≥ 2.6/mL: delivery 23^+3^ weeks (10 days from diagnosis to delivery)	Three patients CRP ≥ 3.9 mg/L and IL-6 < 2.6 ng/dL. Delivery at 26^+0^, 29^+4^ and 37^+0^ weeks, with 29, 90, and 94 days from diagnosis to delivery, respectively	

## Data Availability

The authors can provide additional information regarding the database upon request.

## Abbreviations

Intra-amniotic inflammation (IAI), c-reactive protein (CRP), preterm birth (PTB), interleukin-6 (IL-6).
